# Fertility desires and unmet need for family planning among HIV infected individuals in two HIV clinics with differing models of family planning service delivery

**DOI:** 10.1186/s12905-014-0158-x

**Published:** 2015-01-28

**Authors:** Rhoda K Wanyenze, Joseph KB Matovu, Moses R Kamya, Nazarius M Tumwesigye, Maria Nannyonga, Glenn J Wagner

**Affiliations:** Makerere University School of Public Health, Kampala, Uganda; Makerere University School of Medicine, Kampala, Uganda; Nsambya Home Care HIV Clinic, Kampala, Uganda; RAND Corporation, Santa Monica, CA USA

**Keywords:** Family planning, Unmet need, Fertility, HIV, PMTCT

## Abstract

**Background:**

Eliminating family planning (FP) unmet need among HIV-infected individuals (PLHIV) is critical to elimination of mother-to-child HIV transmission. We assessed FP unmet need among PLHIV attending two clinics with differing models of FP services. Nsambya Home Care provided only FP information while Mulago HIV clinic provided information and contraceptives onsite.

**Methods:**

In a cross-sectional study conducted between February-June 2011, we documented pregnancies, fertility desires, and contraceptive use among 797 HIV-infected men and women (408 in Mulago and 389 in Nsambya). FP unmet need was calculated among women who were married, unmarried but had sex within the past month, did not desire the last or future pregnancy at all or wished to postpone for ≥ two years and were not using contraceptives. Multivariable analyses for correlates of FP unmet need were computed for each clinic.

**Results:**

Overall, 40% (315) had been pregnant since HIV diagnosis; 58% desired the pregnancies. Of those who were not pregnant, 49% (366) did not desire more children at all; 15.7% wanted children then and 35.3% later. The unmet need for FP in Nsambya (45.1%) was significantly higher than that in Mulago at 30.9% (p = 0.008). Age 40+ compared to 18–29 years (OR = 6.05; 95% CI: 1.69, 21.62 in Mulago and OR = 0.21; 95% CI: 0.05, 0.90 in Nsambya), other Christian denominations (Pentecostal and Seventh Day Adventists) compared to Catholics (OR = 7.18; 95% CI: 2.14, 24.13 in Mulago and OR = 0.23; 95% CI: 0.06, 0.80 in Nsambya), and monthly expenditure > USD 200 compared to < USD40 in Nsambya (OR = 0.17; 95% CI: 0.03, 0.90) were associated with FP unmet need.

**Conclusions:**

More than half of the pregnancies in this population were desired. Unmet need for FP was very high at both clinics and especially at the clinic which did not have contraceptives onsite. Lower income and younger women were most affected by the lack of contraceptives onsite. Comprehensive and aggressive FP programs are required for fertility support and elimination of FP unmet need among PLHIV, even with integration of FP information and supplies into HIV clinics.

## Background

Recently there have been renewed efforts towards ‘virtual elimination’ of mother-to-child HIV transmission (eMTCT) [1,2]. In addition to increasing access to more effective antiretroviral drugs during pregnancy and delivery, achieving eMTCT requires limiting the duration of breastfeeding to 12 months and eliminating the current unmet need for family planning [3-5]. However, this latter component-- supporting individuals living with HIV/AIDS to prevent unintended pregnancies-- is an often neglected approach to preventing HIV transmission to infants [6,7]. Knowledge of HIV positive status has been associated with a desire to limit childbearing in some studies [8]. But there are still gaps in ensuring access to sexual and reproductive health services and rights, including contraceptive use by HIV infected individuals [9-11]. The majority of HIV infected women in sub-Saharan Africa (66- 92%) wish to postpone or limit childbearing but only 20-43% use contraception [12].

Several countries, Uganda inclusive, have initiated programs to ensure increased access to family planning (FP) services for HIV infected individuals, with promising models of integration of FP and HIV services [13-15]. However, implementation challenges still remain in terms of how to operationalise the integration, and various untested models of service delivery are used [11]. Several models of integration of FP services have been implemented including integration of FP counseling with onsite contraceptives and FP counseling or information alone, with referral to other sites for contraceptives. Specifically, policies of some faith-based HIV clinics do not allow provision of contraceptives onsite. The impact of onsite and offsite delivery of contraceptives on the uptake of FP and the number of unplanned pregnancies among people living with HIV/AIDS (PLHIV) is not well documented. Also, studies that have evaluated contraceptive use among PLHIV have largely focused on uptake, without consideration for fertility desires and intentions [12]. Unmet need for FP is a more robust indicator of the contraceptive utilization gaps since it focuses on prevention of unplanned pregnancies among individuals who do not wish to have children at all and those who wish to postpone pregnancy for two or more years [16].

In this study, we assessed the fertility desires and the unmet need for FP as well as the clinic specific correlates of unmet need in two HIV clinics with different models of FP integration. Mulago HIV clinic has fully integrated FP services and provides various contraceptives onsite. Nsambya Home Care (NHC), a Catholic founded site has integrated FP information but does not provide contraceptives on site nor formal referral to other facilities for contraception. Understanding the impact of these models on the patterns of contraceptive use and unmet need for FP is important in informing the process of integration of FP services into HIV care and treatment services.

## Methods

### Study setting

Mulago HIV clinic provides care to over 10,000 HIV infected adults. The clinic provides FP education to clients in groups and one-on-one counseling sessions. Mulago also provides onsite contraceptives including male condoms, pills and injectables, and refers patients to the FP clinic within Mulago hospital for other contraceptive options such as implants, intrauterine devices and tubal ligation. NHC, a faith-based (Catholic-supported) HIV clinic, provides care to over 9,000 HIV infected children and adults. NHC also provides information on FP, which is delivered by counselors as part of the one-on-one prevention information and counseling, but does not provide contraceptives at the clinic and has no formal referral linkages for patients who wish to access contraceptives. Both clinics are located in the capital city of Kampala. The Mulago HIV clinic is located within Mulago hospital, the national referral and teaching hospital while NHC is located within Nsambya hospital, a faith based private not for profit hospital.

### Study design and sample size

This was a comparative cross-sectional study. Structured interviews (with pre-determined categories) were conducted with sexually active PLHIV to assess fertility desires and determinants of unmet need for FP. A sample size of 387 respondents per site was calculated, assuming a difference of 10% in uptake (current use) of FP (62% in Mulago and 52% in Nsambya), a power of 80% and alpha of .05 [17,18]. Overall, we interviewed 797 respondents including 408 individuals at NHC and 389 at Mulago HIV clinic between February and June 2011.

### Participant selection and data collection

Individuals who were ≥18 years, had attended the clinic for at least six months, and were sexually active (had sexual intercourse within 12 months) were eligible for participation in the study. At each study site, a systematic sampling technique was applied to select clients from the daily registration list (every sixth client selected). A brief screening tool was used to assess eligibility (age, sexually active, and duration in care).

### Measures

Data were collected on socio-demographic (age, education, marital status, monthly household expenditure, occupation, and religion [Catholic, Protestant, Muslim, other Christian denominations including Pentecostal and Adventists]; and behavioral characteristics (number of sexual partners in the past 12 months, condom use in the past 12 months, HIV status disclosure), whether or not participants were on antiretroviral drugs (ARVs) and duration on ARVs, perceived health status (Poor/fair; Good; and Very good), CD4 cell count at last test (<350, 350–499, 500+ and unknown) and partner HIV status (HIV status unknown; known HIV-negative; and known HIV-positive). Participants were asked if they (females) or their partners (males) had ever been pregnant since HIV-positive diagnosis, and those reporting pregnancy were asked if they desired the last pregnancy (then; later; or not at all). Participants were also asked if they or their partners were currently pregnant; and if not, they were asked about desire for future pregnancy (coded as ‘then/immediately’ , ‘later’; ‘not at all’ , or don’t know’). Participants were also asked about their partners’ desire for more children and whether they discussed with their partners the number of children to have and when to have them.

FP methods included both modern and traditional FP methods. Modern FP methods included male/female sterilization, male/female condoms, intra-uterine device, pills, injectables, implants, foam/jelly, diaphragm and emergency contraception. We also defined effective FP methods to include current use of any modern FP methods with the exception of inconsistent condom use. Inconsistent use of condoms is not very effective as a FP tool since it would require understanding and timing of the fertile period [19,20]. Participants were asked several questions in relation to the use of FP methods; whether or not they were currently using any FP method, and if so, what type of method. Participants who said they used condoms were asked about the frequency of condom use in the past 12 months, which was categorized as never, sometimes, always (consistent), and don’t know (not sure). At analysis, the participants who never used condoms, used sometimes and those who were not sure, were categorized as inconsistent condom users.

Unmet need for FP was defined as the proportion of all married women and unmarried women who had sex within four weeks prior to the interview who: (a) did not desire to have the last pregnancy or desired that pregnancy at another time; or (b) were not currently pregnant but did not desire to have a future pregnancy or desired to wait for two or more years; and (c) were not using an effective method of family planning [19,20]. Unmet need for FP was stratified into: (i) unmet need for limiting childbirth; and (ii) unmet need for child spacing. Women were considered to have an unmet need for limiting childbirth if they did not desire to have the last pregnancy at all or did not (at all) want to have any future pregnancy but were not using an effective family planning method. On the other hand, women were considered to have an unmet need for child spacing if they desired to have their last or future pregnancy two or more years later but were not currently using an effective FP method [16].

### Statistical analyses

We computed descriptive statistics to describe the socio-demographic and behavioral characteristics between the two clinics. We used Chi Square tests to detect differences in the descriptive variables reported in the two clinics. We further conducted univariate analysis to generate statistics on participant characteristics, pregnancy status, fertility desire and current use of FP methods stratified by HIV clinic and gender. In the general description of FP use, we included FP methods that the men reported that their sexual partners were using. However, based on the definition for FP unmet need, only women who were married or in consensual relationship were included at bivariate and multivariate analysis for correlates of unmet need [16]. The unmet need for FP was compared between the two clinics, using Pearson’s Chi Square tests. Potential correlates included in the bivariate and multivariate analysis were socio-demographic, health status, and behavioral characteristics. In the initial analysis, we collapsed the data across the two clinics into a combined model that included the site (Nsambya and Mulago) as a covariate and the site was the only significant correlate of FP unmet need. Due to the differences in the FP service delivery approaches and thus potential differences in correlates across sites, we conducted bivariate and multivariate analyses separately for each HIV clinic in order to identify which factors were driving the unmet need at each clinic. Only results from the clinic-specific analyses are reported. Variables were considered to be significantly associated with unmet need for FP if they had a p-value less than 0.05 (P < 0.05).

### Ethics statement

Written informed consent was sought from eligible clients before administration of the survey, which was conducted in a private setting. The study was approved by Makerere University School of Public Health Higher Degrees Research and Ethics committee and the Uganda National Council for Science and Technology. Permission to conduct the study was also sought from Mulago HIV clinic and Nsambya Home Care (NHC) HIV clinic management.

## Results

Overall 942 clients were screened for participation; 140 were not eligible while 797 of the 802 who were eligible (99.3%) accepted to participate and were enrolled into the study, including 408 in Mulago and 389 in Nsambya. Table 1 shows the socio-demographic and behavioral characteristics of the participants, stratified by HIV clinic and gender. The majority of the participants were aged 30–39 years (52.5%), had primary (48.7%) or higher (46.4%) education, were currently married (68.0%), and belonged to the Catholic (40.3%) or Protestant (32.9%) religion. Other Christian denominations constituted 13% (104), and 83.6% of these (87) were Pentecostal while the rest were Seventh Day Adventists (SDA). In terms of household expenditure, the majority (74.7%) spent Uganda Shillings (UGX) 100,000 – 500,000 [between US$40-200] as monthly household expenditure and were largely engaged in commercial activities (46.7%). Most participants reported one sexual partner in the past year (82.8%), nearly half (47.4%) had partners with known HIV-positive status and three-quarters (74.5%) reported disclosing their HIV status to the partner. The majority (79.3%) were currently enrolled on ARVs, 32.0% had a CD4 cell count <350 cells/μL at last test while 44.9% rated their health status as very good. For some variables, there were statistically significant differences across the two clinics (education level, marital status, monthly expenditure, occupation, number of sexual partners, percentage and duration on ARVs, and perceived health status) (Table 1).

**Table 1:** **Socio-demographic and behavioral characteristics of HIV infected clients receiving care at Mulago and Nsambya HIV clinics in Kampala, Uganda**

**Characteristic**	**Mulago (N = 408)**		**Nsambya home care (N = 389)**		**Total (N = 797)**	**P-value** ^**a**^
	**Males (156)**	**Females (252)**	**Males (129)**	**Females (260)**		
**Age-group**						0.18
18-29	16 (10.3)	94 (37.3)	10 (7.8)	73 (28.1)	193 (24.2)	
30-39	85 (54.5)	120 (47.6)	57 (44.2)	156 (73.2)	418 (52.5)	
40+	55 (35.2)	38 (15.10)	62 (48.1)	31 (11.9)	186 (23.3)	
**Education**						0.001
None	03 (1.9)	21 (8.3)	04 (3.1)	11 (4.2)	39 (4.9)	
Primary	88 (56.4)	133 (52.8)	60 (46.5)	107 (41.1)	388 (48.7)	
Secondary	49 (31.4)	82 (32.5)	49 (38.0)	107 (41.1)	287 (36.0)	
Tertiary	16 (10.3)	16 (6.4)	16 (12.4)	35 (13.5)	83 (10.4)	
**Marital status**						0.003
Single	10 (6.4)	24 (9.5)	08 (6.2)	38 (14.6)	80 (10.0)	
Currently married	121 (77.6)	154 (61.1)	103 (79.8)	164 (63.1)	542 (68.0)	
Divorced/separated	21 (13.4)	57 (22.6)	11 (8.5)	32 (12.3)	121 (15.2)	
Widowed	04 (2.6)	17 (6.8)	07 (5.4)	26 (10.0)	54 (6.8)	
**Religion**						0.74
Catholic	63 (40.4)	99 (39.3)	54 (41.9)	105 (40.4)	321 (40.3)	
Protestant	64 (41.0)	74 (29.4)	49 (38.0)	75 (28.8)	262 (32.9)	
Muslim	17 (10.9)	42 (16.7)	12 (9.3)	39 (15.0)	110 (13.8)	
Other Christian	12 (7.7)	37 (14.6)	14 (10.8)	41 (15.8)	104 (13.0)	
**Average monthly expenditure**						<0.0001
<100,000	11 (7.1)	23 (9.1)	14 (10.8)	47 (18.1)	95 (11.9)	
100,000 – 500,000	110 (70.5)	191 (75.8)	106 (82.2)	188 (72.3)	595 (74.7)	
>500,000	35 (22.4)	38 (15.1)	09 (7.0)	25 (9.6)	107 (13.4)	
**Occupation**						0.003
Salaried	24 (15.4)	33 (13.1)	26 (20.2)	42 (16.2)	125 (15.7)	
Business/commercial	55 (35.3)	117 (46.4)	67 (51.9)	133 (15.2)	372 (46.7)	
Casual worker	48 (30.8)	34 (13.5)	26 (20.2)	30 (11.5)	138 (17.3)	
Other employment	29 (18.5)	68 (27.0)	10 (7.7)	55 (21.1)	162 (20.3)	
**Number of sexual partners (past 12 months)**						<0.0001
1	101 (64.7)	210 (83.3)	107 (83.0)	242 (93.1)	660 (82.8)	
2+	55 (35.3)	42 (16.7)	22 (17.0)	18 (6.9)	137 (17.2)	
**Condom use (past 12 months)**						0.14
Never	13 (8.3)	64 (25.4)	14 (10.9)	40 (15.4)	131 (16.4)	
Sometimes	55 (35.3)	111 (44.0)	45 (34.9)	116 (44.6)	327 (41.0)	
Always	88 (56.4)	77 (30.6)	70 (54.2)	104 (40.0)	339 (42.5)	
**Partner HIV status**						0.07
Unknown	41 (26.3)	117 (46.4)	31 (24.0)	95 (36.5)	284 (35.6)	
Known HIV negative	32 (20.5)	27 (10.7)	37 (28.7)	39 (15.0)	135 (16.9)	
Known HIV positive	83 (53.2)	108 (42.9)	61 (47.3)	126 (48.5)	378 (47.4)	
**HIV status disclosure to partner**						0.07
No	29 (18.6)	86 (34.1)	22 (17.0)	66 (25.4)	203 (25.5)	
Yes	127 (81.4)	166 (65.9)	107 (83.0)	194 (74.6)	594 (74.5)	
**Currently on ARVs**						<0.0001
No	35 (22.4)	89 (35.3)	08 (6.2)	33 (12.7)	165 (20.7)	
Yes	121 (77.6)	163 (64.7)	121 (93.8)	227 (87.3)	632 (79.3)	
**Duration since initiated ARVs** ^**b**^						<0.0001
<1 year	25 (20.7)	28 (17.2)	15 (12.4)	45 (19.8)	114 (17.9)	
1 year	16 (13.2)	33 (20.2)	18 (14.9)	42 (18.5)	109 (17.2)	
2 years	36 (29.8)	36 (22.1)	21 (17.4)	25 (11.0)	118 (18.7)	
3+ years	44 (36.3)	66 (40.5)	67 (55.3)	115 (50.7)	292 (46.2)	
**CD4 cell count (last time tested)**						<0.0001
<350	55 (35.2)	69 (27.4)	51 (39.5)	79 (30.4)	254 (31.9)	
350-499	24 (15.4)	47 (18.6)	46 (35.7)	78 (30.0)	195 (24.4)	
500+	34 (21.8)	57 (22.6)	12 (9.3)	85 (32.7)	188 (23.6)	
Unknown	43 (27.6)	79 (31.4)	20 (15.5)	18 (6.9)	160 (20.1)	
**Health status**						0.001
Poor/Fair	27 (17.3)	59 (23.4)	22 (17.0)	23 (8.9)	131 (16.4)	
Good	66 (42.3)	89 (35.3)	50 (38.8)	103 (39.6)	308 (38.6)	
Very Good	63 (40.4)	104 (41.3)	57 (44.2)	134 (51.5)	358 (44.9)	

^a^Pearson Chi Square. ^b^Expressed out of those who were currently taking ARVs. ARVs, antiretroviral drugs.

### Pregnancy status and fertility desires

Table 2 shows the pregnancy status and fertility desires of the respondents, stratified by gender and HIV clinic. Overall, 39.5% of the participants reported that they or their partners had ever been pregnant since HIV diagnosis; 35.6% of these had been pregnant or their partners had been pregnant two or more times. Of those who had ever been pregnant, 25.4% reported that they did not desire the last pregnancy at all, while 16.5% desired the pregnancy later. Although not statistically significant, a higher percentage of women in Nsambya (36%) did not desire their last pregnancy at all, compared to those in Mulago (25.7%). Only 6.3% of all participants reported that they or their partners were currently pregnant. Half of those that were not currently pregnant (49%) did not desire or were not sure if they desired any future pregnancy. A higher percentage of women in Nsambya reported the desire for limiting pregnancy compared to Mulago. In Nsambya, half of the women (50.2%) did not desire any more children at all, compared to 41.6% in Mulago. Several participants reported that their partners desired more children at the time (33.0%) or in the future (18.6%). Sixty two per cent reported that they discussed with their partners about the number of children that they should have while 50.6% reported that they discussed with their partners about when to have children.

**Table 2:** **Pregnancy status and fertility desires among HIV infected clients receiving care Mulago and Nsambya HIV clinics in Kampala, Uganda**

**Variable**	**Mulago (N = 408)**		**Nsambya home care (N = 389)**		**Total (N = 797)**	**P-value** ^**a**^
	**Males (156)**	**Females (252)**	**Males (129)**	**Females (260)**		
**Pregnant/partner pregnant since HIV diagnosis**						0.64
No/Not sure	111 (71.2)	139 (55.2)	83 (64.3)	149 (57.3)	482 (60.5)	
Yes	45 (28.8)	113 (44.8)	46 (35.7)	111 (42.7)	315 (39.5)	
**Number of times pregnant/partner pregnant** ^**a**^						0.37
Once	31 (68.9)	67 (59.3)	33 (71.7)	72 (64.9)	203 (64.4)	
2+ times	14 (31.1)	46 (40.7)	13 (28.3)	39 (35.1)	112 (35.6)	
**Desired/partner desired last pregnancy** ^**b**^						<0.0001
Yes, then	30 (66.7)	52 (46.0)	37 (80.4)	64 (57.7)	183 (58.1)	
Yes, but later	09 (20.0)	32 (28.3)	04 (8.7)	07 (6.3)	52 (16.5)	
Not at all	06 (13.3)	29 (25.7)	05 (10.9)	39 (36.0)	80 (25.4)	
**Currently pregnant/partner currently pregnant**						0.91
No	149 (95.5)	233 (92.5)	124 (96.1)	241 (92.7)	747 (93.7)	
Yes	07 (4.5)	19 (7.5)	05 (3.9)	19 (7.3)	50 (6.3)	
**Desire/partner desires future pregnancy** ^**c**^						0.17
Yes, now	18 (12.1)	37 (15.9)	24 (19.3)	38 (15.8)	117 (15.7)	
Yes, but later	48 (32.2)	99 (42.5)	35 (28.3)	82 (34.0)	264 (35.3)	
Not at all/Don’t know	83 (55.7)	97 (41.6)	65 (52.4)	121 (50.2)	366 (49.0)	
**Partner desire for more children**						0.005
Yes, now	25 (16.0)	103 (40.9)	36 (27.9)	99 (38.1)	263 (33.0)	
Yes, but later	39 (25.0)	41 (16.3)	24 (18.6)	44 (16.9)	148 (18.6)	
Not at all	45 (28.9)	46 (18.2)	51 (39.5)	66 (25.4)	208 (26.1)	
Don’t know	47 (30.1)	62 (24.6)	18 (14.0)	51 (19.6)	178 (22.3)	
**Discussed with partner about number of children to have**						0.002
No	63 (40.4)	115 (45.6)	31 (24.0)	98 (37.7)	307 (38.5)	
Yes	93 (59.6)	137 (54.4)	98 (76.0)	162 (62.3)	490 (61.5)	
**Discussed with partner about when to have children**						0.03
No	77 (49.4)	139 (55.2)	45 (34.9)	131 (50.4)	392 (49.2)	
Yes	79 (50.6)	113 (44.8)	84 (65.1)	129 (49.6)	405 (50.8)	

^a^Pearson Chi Square.

^b^Expressed out of women who have ever been pregnant/men whose partners have ever been pregnant since HIV-positive diagnosis.

^c^Expressed out of women who were not currently pregnant/men whose partners were not currently pregnant.

### Current use of family planning methods and unmet need for family planning

Table 3 shows the proportion of men and women reporting current use of FP in Mulago and Nsambya HIV clinics, stratified by gender. Overall, 80.5% of the participants reported that they were currently using any FP method while 77.9% reported that they were currently using a modern method of FP. However, only 58.2% were using an effective modern method of FP, with lower proportions reported among women in Nsambya (50%) than in Mulago (57.9%); p = 0.04. The use of less effective FP methods among women in Nsambya was largely due to more prevalent inconsistent condom use. Among those who used condoms in Nsambya, 69.4% reported inconsistent use compared to 60.0% in Mulago. Compared to Mulago, a larger proportion of women in Nsambya also reported using withdrawal (16.3% vs. 4.9%; p = 0.002) and rhythm methods (14.9% vs. 5.3%; p = 0.03). In both clinics, current use of FP methods was higher among men than women, largely driven by use of male condoms. In Mulago, current use of any FP method was 92.3% among men compared to 72.6% among women. Likewise, in Nsambya, current use of any FP method was 86.8% compared to 77.7% among women.

**Table 3:** **Current use of family planning services and unmet need among HIV infected women** ^**a**^ **receiving care at Mulago and Nsambya HIV clinics in Kampala, Uganda**

**Variable**	**Mulago (N = 408)**		**Nsambya home care (N = 389)**		**Total (N = 797)**	**P-value** ^**b**^
	**Males (156)**	**Females (252)**	**Males (129)**	**Females (260)**		
**Currently using any family planning method**						0.84
No	12 (7.7)	69 (27.4)	17 (13.2)	58 (22.3)	156 (19.6)	
Yes	144 (92.3)	183 (72.6)	112 (86.8)	203 (77.7)	641 (80.5)	
**Currently using any modern** ^**c**^ **family planning method**						0.85
No	14 (9.0)	75 (29.8)	19 (14.7)	68 (26.2)	176 (22.1)	
Yes	142 (91.0)	177 (70.2)	110 (85.3)	192 (73.8)	621 (77.9)	
**Currently using an effective** ^**d**^ **modern family planning method**						0.04
No	50 (32.0)	106 (42.1)	47 (36.4)	130 (50.0)	333 (41.8)	
Yes	106 (68.0)	146 (57.9)	82 (63.6)	130 (50.0)	464 (58.2)	
**Overall unmet need for family planning**						0.008
No	-	114 (69.1)	-	90 (54.9)	204 (62.0)	
Yes	-	51 (30.9)	-	74 (45.1)	125 (38.0)	
**Unmet need for limiting child birth**						0.20
No	-	56 (68.3)	-	59 (59.0)	115 (63.2)	
Yes	-	26 (31.7)	-	41 (41.0)	67 (36.8)	
**Unmet need for child spacing**						0.008
No	-	58 (69.9)	-	31 (48.4)	89 (60.5)	
Yes	-	25 (30.1)	-	33 (51.6)	58 (39.5)	

^a^Among women aged 18–49 years.

^b^Pearson Chi Square.

^c^Modern family planning methods included: male/female sterilization, male/female condoms, intra-uterine device (IUD), pills, injectables, implants, foam/jelly, diaphragm, and emergency contraception.

^d^Effective modern family planning methods included all the modern methods minus inconsistent condom use.

**Figure 1:**
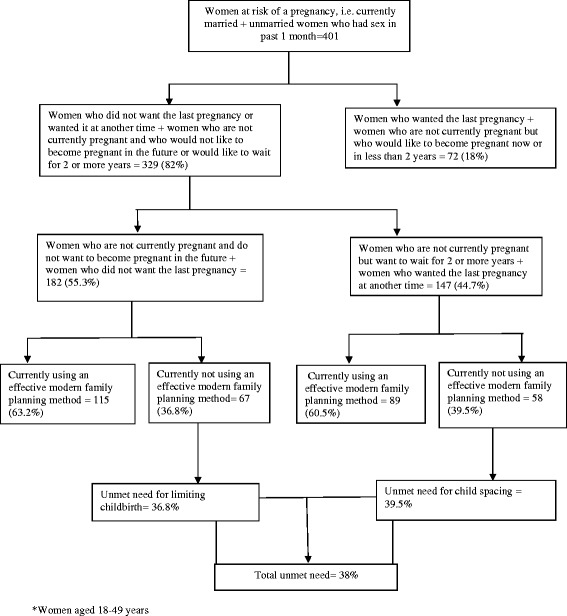
Unmet need for family planning among 329 HIV women* receiving care at Mulago and Nsambya HIV clinics in Kampala, Uganda.

Figure 1 shows the proportion of women who had unmet need for FP at both clinics. There were 401 women who were currently married or unmarried but had sex in the past four weeks (among the 194 women who were not married, 42.8% had sex in the past month). Of the 401 women, 82.0% (329) either did not want the last pregnancy or wanted it at another time. Of these, 55.3% (182) did not want their last pregnancy or any pregnancy in the future while 44.7% (147) wanted to wait for two or more years. Of the 182 women who did not want their last or future pregnancy, 36.8% were not using any effective FP method. Likewise, of the 147 women who wanted their last pregnancy after two or more years, 39.5% were not using any effective FP method. Thus, 36.8% of the women had an unmet need for limiting childbirth while 39.5% had an unmet need for child spacing, contributing to an overall unmet need of 38%.

As shown in Table 3, overall unmet need was higher in Nsambya (45.1%) than in Mulago (30.9%) (p = 0.008). The difference in the unmet need for limiting childbirth in Nsambya (41%) and Mulago (31.7%) was not statistically significant. However, the unmet need for child spacing in Nsambya (51.6%) was significantly higher than that in Mulago (30.1%) (p = 0.008). While the unmet need for limiting and spacing was similar in Mulago; in Nsambya, the unmet need for child spacing (51.6%) was much higher than the unmet need for limiting childbirth (41.0%).

### Correlates of unmet need for family planning in Mulago and Nsambya

Table 4 shows the unadjusted and adjusted site specific odds ratio (OR) and 95% confidence intervals (95%CI) associated with unmet need for FP among HIV-positive women at Mulago and Nsambya. In Mulago, at the bivariate analysis, the factors associated with unmet need for FP were age-group 40+ years compared to 18–29 years (OR = 3.67; 95% CI: 1.35, 9.96) and belonging to the Pentecostal and Adventists Christian denominations compared to Catholics (OR = 4.47; 95% CI: 1.65, 12.11). After the adjusted analyses, age and religious affiliation remained significantly associated with unmet need for FP in Mulago [age-group 40+ years compared to18-29 years: Adjusted [Adj.] OR = 6.05; 95% CI: 1.69, 21.62; belonging to the Pentecostal/Adventists religious affiliation (in comparison to the Catholic religion): OR = 7.18; 95%CI: 2.14, 24.13]. Unmet need for FP was not associated with current enrolment on ARVs, CD4 cell count at last test, and health status, among other factors.

**Table 4:** Unadjusted and adjusted odds ratios and 95% confidence intervals associated with unmet need among 329 HIV infected women* receiving care at Mulago and Nsambya HIV clinics in Kampala, Uganda

**Characteristic**	**Mulago**		**Nsambya home care**	
	**Unadjusted OR and 95% confidence intervals (CI)**	**Adjusted OR and 95% CI**	**Unadjusted OR and 95% CI**	**Adjusted OR and 95% CI**
**Age-group**				
18-29	1.00	1.00	1.00	1.00
30-49	1.67 (0.76, 3.68)	1.68 (0.63, 4.48)	0.56 (0.28, 1.10)	0.51 (0.22, 1.89)
40+	3.67 (1.35, 9.96)	6.05 (1.69, 21.62)	0.39 (0.12, 1.32)	0.21 (0.05, 0.90)
**Education**				
None	1.00	1.00	1.00	1.00
Primary	1.16 (0.29, 4.66)	0.57 (0.11, 2.85)	0.98 (0.20, 4.74)	0.82 (0.10, 6.66)
Secondary	1.75 (0.42, 7.19)	1.11 (0.20, 6.13)	1.37 (0.29, 6.53)	1.52 (0.19, 12.18)
Tertiary	1.29 (0.20, 8.43)	0.74 (0.08, 6.48)	0.53 (0.80, 3.54)	0.44 (0.04, 5.16)
**Marital status**				
Single	1.00	1.00	1.00	1.00
Currently married	1.28 (0.38, 4.33)	1.19 (0.26, 5.48)	1.86 (0.45, 7.73)	5.80 (0.95, 35.26)
Divorced/separated	0.66 (0.14, 3.01)	0.92 (0.15, 5.53)	0.50 (0.62, 4.00)	1.36 (0.12, 15.75)
Widowed	0.50 (0.04, 5.74)	0.45 (0.03, 6.51)	1.33 (0.20, 8.71)	3.26 (0.36, 29.38)
**Religion**				
Catholic	1.00	1.00	1.00	1.00
Protestant	2.13 (0.93, 4.86)	2.27 (0.90, 5.72)	0.99 (0.46, 2.11)	0.95 (0.37, 2.44)
Muslim	1.26 (0.42, 3.77)	1.65 (0.48, 5.65)	1.29 (0.54, 3.06)	1.22 (0.42, 3.52)
Other Christian (Pentecostals and Adventists)	4.47 (1.65, 12.11)	7.18 (2.14, 24.13)	0.40 (0.14, 1.14)	0.23 (0.06, 0.80)
**Average monthly household expenditure**				
<100,000	1.00	1.00	1.00	1.00
100,000 – 500,000	6.11 (0.77, 48.37)	5.51 (0.62, 48.73)	0.48 (0.19, 1.17)	0.50 (0.16, 1.58)
>500,000	7.94 (0.91, 69.43)	7.68 (0.72, 82.08)	0.23 (0.06, 0.86)	0.17 (0.03, 0.90)
**Currently on ARVs**				
No	1.00	1.00	1.00	1.00
Yes	1.54 (0.75, 3.18)	2.28 (0.95, 5.46)	0.87 (0.37, 2.05)	1.57 (0.53, 4.69)
**CD4 cell count at last test**				
<350	1.00	1.00	1.00	1.00
350-499	0.88 (0.34, 2.33)	0.77 (0.23, 2.50)	1.73 (0.78, 3.84)	1.87 (0.71, 4.88)
500+	1.11 (0.43, 2.83)	1.06 (0.34, 3.29)	1.13 (0.50, 2.55)	1.59 (0.60, 4.22)
Unknown	0.98 (0.41, 2.34)	0.79 (0.26, 2.39)	1.33 (0.38, 4.61)	1.24 (0.26, 5.87)
**Partner HIV status**				
Unknown	1.00	1.00	1.00	1.00
Known HIV-negative	1.03 (0.29, 3.69)	1.05 (0.20, 5.53)	0.96 (0.36, 2.61)	0.34 (0.08, 1.44)
Known HIV-positive	1.31 (0.65, 2.64)	1.37 (0.40, 4.72)	1.50 (0.76, 2.98)	0.76 (0.24, 2.37)
**Health status**				
Poor/Fair	1.00	1.00	1.00	1.00
Good	0.99 (0.41, 2.38)	0.58 (0.19, 1.80)	1.72 (0.56, 5.33)	1.19 (0.31, 4.61)
Very Good	0.85 (0.36, 2.01)	0.54 (0.19, 1.56)	1.23 (0.41, 3.70)	0.89 (0.23, 3.36)
**Number of sexual partners in past 12 months**				
1	1.00	1.00	1.00	1.00
2+	0.80 (0.34, 1.88)	0.97 (0.34, 2.71)	0.18 (0.04, 0.83)	0.19 (0.03, 1.19)
**HIV status disclosure to partner**				
No	1.00	1.00	1.00	1.00
Yes	1.27 (0.59, 2.73)	0.50 (0.13, 2.00)	1.85 (0.83, 4.13)	1.10 (0.29, 4.20)
**Belief that an HIV+ mother can have an HIV-free baby**				
No	1.00	1.00	1.00	1.00
Yes	1.35 (0.14, 13.31)	1.92 (0.14, 26.84)	2.12 (0.40, 11.24)	3.00 (0.22, 40.94)
**Belief that mother-to-child transmission can be avoided**				
No	1.00	1.00	1.00	1.00
Yes	0.89 (0.21, 3.70)	0.57 (0.11, 2.95)	1.25 (0.34, 4.61)	0.44 (0.05, 3.64)

*Among women aged 18–49 years.

In Nsambya, at the bivariate analysis, the factors associated with unmet need for FP were average monthly household expenditure > UGX 500,000 [>$200] compared to expenditure of <100,000 UGX [$40] (OR = 0.23; 95% CI: 0.06, 0.86) and reporting 2 or more sexual partners (compared to one partner) in the past 12 months (OR = 0.18; 95% CI: 0.04, 0.83). At multivariate analysis, the factors that remained independently associated with unmet need for FP were: age-group 40+ years (Adj. OR = 0.21; 95% CI: 0.05, 0.90) and belonging to Pentecostal/Adventist religions (Adj. OR = 0.23; 95% CI: 0.06, 0.80). Whereas the Pentecostal/Adventists were more likely to have unmet need in Mulago, in Nsambya the Pentecostal/Adventists were less likely to have unmet need for FP in comparison to the Catholics. Additionally, an average monthly household expenditure > UGX 500,000 reduced the likelihood for FP unmet need (Adj. OR = 0.17; 95%CI: 0.03, 0.90). Unmet need for FP in Nsambya was not associated with ARVs, CD4 cell count at last test, and health status, among other factors.

## Discussion

This study assessed fertility desires among HIV infected men and women as well as unmet need for FP among the women in two HIV clinics with differing models of FP service integration. Overall, a significant proportion of men and women (40%) at both clinics had been pregnant since their HIV diagnosis and more than half desired these pregnancies then. Among those who were not pregnant, 49% did not desire to have any more children at all; yet, half of these were not using effective FP methods. The overall unmet need for FP was very high, at 38%.

This study shows that a large number of pregnancies among PLHIV are desired [21,22]. With improved access to ART and survival as well as expansion of PMTCT services, more PLHIV are likely to desire children [23-25]. Yet, most FP services for PLHIV focus predominantly on contraception with limited support for PLHIV who wish to have children [21,26,27]. To ensure the SRH needs and rights of PLHIV, FP services should cater for both the PLHIV who want children and those who wish to delay or limit childbearing.

The use of FP methods among PLHIV may be quite high, but many are not using effective FP methods and thus remain at risk of getting pregnant [19,27]. With about half of the women not desiring any children at all, long-term or irreversible contraceptives would be ideal for this group [7,28]. However, use of ineffective methods was common in both sites and most prominent at Nsambya, which did not have onsite contraceptives. Studies have reported the use ineffective FP methods among PLHIV, with overreliance on condoms which are not effective for contraception especially when used inconsistently [12,27-29]. The overreliance on condoms among PLHIV is largely due to the need to reduce HIV transmission; dual contraception and HIV prevention can be enhanced through consistent condom use and use of condoms with an additional effective contraceptive method [6,11].

After accounting for the fertility desires, this study demonstrates a very high unmet need for FP. At 38%, the overall unmet need in this population is close to the 34.5% in the general population in Uganda [30]. However, this gap is a major hindrance to the eMTCT efforts [4,5]. Not surprisingly, the unmet need for FP was higher in Nsambya (45%), which provided only FP information with no formal referral mechanisms for contraceptives, compared to Mulago (31%) which had integrated both FP information and contraceptives onsite. More women in Nsambya reported inconsistent condom use and rhythm methods. However, the high unmet need could also be attributed to gaps in counseling at both clinics since the unmet need in Mulago was also high despite the integration of more comprehensive FP services. The absence of FP supplies onsite at Nsambya further affected the unmet need and especially the unmet need for spacing.

Religion influenced FP use; in comparison to the Catholic women, the Pentecostal/Adventists were more likely to have unmet need at Mulago while the Pentecostal were less likely to have unmet need than the Catholic women at Nsambya. Religion may influence social behaviors including contraceptive use and unmet need for FP; however, we cannot fully explain the variance in the unmet need for FP among Pentecostals/Adventists between the two clinics [31]. In both facilities, older women were more likely to have unmet need, while women with higher monthly expenditure at Nsambya were less likely to have unmet need. Older women had a higher unmet need for FP probably due to lower perception of the risk of pregnancy, and would require enhanced FP counseling support. These findings also show that the absence of contraceptives at the site is more likely to affect the women with lower income. Generally, HIV infected women prefer to get their contraceptives within the HIV clinics for various reasons including convenience, provider expertise, a sense of belonging, and privacy concerns [29,32]. What we cannot fully explain from these findings is why the unmet need for FP in Mulago, although lower than Nsambya, remains very high at 31% when information and contraceptives as well as referral for permanent methods are available within the same hospital. This remaining gap might be attributed to other factors such as availability of preferred FP methods and fear of side effects of contraceptives, among others [28].

This study has some limitations. This study did not assess the quality of FP counseling at the clinics and the potential impact of provider stigma and judgmental attitudes on FP unmet need. However, from our previous studies, such stigma was more prominent towards PLHIV who wanted children rather than those who wanted to delay or limit childbearing [27]. Also, the formula that was used for calculation of unmet need is one that is commonly used for community based surveys and was not designed for facility based studies such as ours. However, the discounting of women who desired more children within two years in the estimation of the FP gap remains valid for this population.

## Conclusions

This study shows prominent fertility desires among PLHIV as well as a very high unmet need for FP among HIV infected women, especially those who did not have contraceptives within their HIV clinic. The study highlights a need to enhance FP counseling and aggressively address the unmet need among HIV infected women, even with integration of FP information and supplies into HIV clinics. The findings also underscore the need for a balanced approach to FP that addresses the needs of those who wish to have more children.

## Abbreviations

eMTCT: Virtual elimination’ of mother-to-child transmission
FP: Family planning
HIV: Human immunodeficiency virus
NHC: Nsambya Home Care
PLHIV: People living with HIV
PMTCT: Prevention of mother-to-child transmission
SDA: Seventh Day Adventist

## References

[1] World Health Organization. Antiretroviral drugs for treating pregnant women and preventing HIV infection in infants: recommendations for a public health approach. – 2010 version. 2010. World Health Organization, 20 Avenue Appia, 1211 Geneva 27, Switzerland. 26180894

[2] World Health Organization. Consolidated guidelines on general HIV care and the use of antiretroviral drugs for treating and preventing HIV infection: recommendations for a public health approach. 2013. World Health Organization, 20 Avenue Appia, 1211 Geneva 27, Switzerland.

[3] Hladik W, Stover J, Esiru G, Harper M, Tappero J (2009). The contribution of family planning towards the prevention of vertical HIV transmission in Uganda. PLoS One.

[4] Mahy M, Stover J, Kiragu K, Hayashi C, Akwara P, Luo C (2010). What will it take to achieve virtual elimination of mother-to-child transmission of HIV? An assessment of current progress and future needs. Sex Transm Infect.

[5] Ciaranello AL, Perez F, Keatinge J, Park JE, Engelsmann B, Maruva M (2012). What will it take to eliminate pediatric HIV? Reaching WHO target rates of mother-to-child HIV transmission in Zimbabwe: a model-based analysis. PLoS Med.

[6] Leach-Lemens C. Preventing unintended pregnancies in women living with HIV in resource-poor settings. HIV & AIDS Treatment in Practice. Issue 155, March 2010. http://www.aidsmap.com/Preventing-unintended-pregnancies-in-women-living-with-HIV-in-resource-poor-settings/page/1396517/.

[7] Kancheva Landolt N, Ramautarsing RA, Phanuphak N, Teeratakulpisarn N, Pinyakorn S, Rodbamrung P (2013). Factors associated with the use of irreversible contraception and continuous use of reversible contraception in a cohort of HIV-positive women. Contraception.

[8] Johnson KB, Akwara P, Rutstein SO, Bernstein S (2009). Fertility preferences and the need for contraception among women living with HIV: the basis for a joint action agenda. AIDS.

[9] Homsy J, Bunnel R, Moore D, King R, Malamba S, Nakityo R (2009). Reproductive intentions and outcomes among women on antiretroviral therapy in rural Uganda: a prospective cohort study. PLoS One.

[10] Warren CE, Abuya T, Askew I, Integra Initiative (2013). Family planning practices and pregnancy intentions among HIV-positive and HIV-negative postpartum women in Swaziland: a cross sectional survey. BMC Pregnancy Childbirth.

[11] Lopez LM, Otterness C, Chen M, Steiner MJ, Gallo MF (2013). Behavioral interventions for improving condom use for dual protection. Cochrane Database Syst Rev.

[12] Sarnquist CC, Rahangdale L, Maldonado Y (2013). Reproductive health and family planning needs among HIV-infected women in Sub-Saharan Africa. Curr HIV Res.

[13] Population Action International 2012. Promoting FP/RH-HIV/AIDS Integration: A Summary of Global Health Initiative Strategies in Ethiopia, Kenya, Tanzania, and Zambia. Available at: http://populationaction.org/wp-content/uploads/2012/07/GHI_Analysis_FINAL.pdf. July 2012 Population Action International 1300 19th Street NW Second Floor; Washington, DC 20036 USA.

[14] Kosgei RJ, Lubano KM, Shen C, Wools-Kaloustian KK, Musick AM, Siika BS (2011). Impact of integrated family planning and HIV care services on contraceptive use and pregnancy outcomes: a retrospective cohort study. J Acquir Immune Defic Syndr.

[15] Stephenson R, Vwalika B, Greenberg L, Ahmed Y, Vwalika C, Chomba E (2011). A Randomized controlled trial to promote long-term contraceptive use among HIV Serodiscordant and concordant positive couples in Zambia. J Womens Health (Larchmt).

[16] Millennium Development Goal Indicators website: http://millenniumindicators.un.org/.

[17] Bland M. An introduction to medical statistics, 3rd Edition. 2000. Oxford University Press 2000, Oxford University Press Inc., New York, USA.

[18] Wanyenze RK, Wagner GJ, Tumwesigye NM, Nannyonga M, Wabwire-Mangen F, Kamya MR (2013). Fertility and contraceptive decision-making and support for HIV infected individuals: client and provider experiences and perceptions at two HIV clinics in Uganda. BMC Public Health.

[19] Cleland J, Ali MM (2004). Reproductive consequences of contraceptive failure in 19 developing countries. Obstet Gynecol.

[20] World Health Organization. Sexual and reproductive health of women living with HIV/AIDS: Guidelines on care, treatment and support for women living with HIV/AIDS and their children in resource-constrained settings. 2006. World Health Organization, 20 Avenue Appia, 1211 Geneva 27, Switzerland.

[21] Wagner G, Linnemayr S, Kityo C, Mugyenyi P (2012). Factors associated with intention to conceive and its communication to providers in Uganda. Matern Child Health J.

[22] Beyeza-Kashesya J, Kaharuza F, Mirembe F, Neema S, Ekstrom AM, Kulane A (2009). The dilemma of safe sex and having children: challenges facing HIV sero-discordant couples in Uganda. Afr Health Sci.

[23] Kaida A, Andia I, Maier M, Strathdee SA, Bangsberd DR, Spiegel J (2006). The potential impact of antiretroviral therapy on fertility in sub-Saharan Africa. Curr HIV/AIDS Rep.

[24] Nattabi B, Li J, Thompson SC, Orach CG, Earnest J (2009). A systematic review of factors influencing fertility desires and intentions among people living with HIV/AIDS: implications for policy and service delivery. AIDS Behav.

[25] Myer L, Carter RJ, Katyal M, Toro P, El-Sadr WM, Abrams EJ (2010). Impact of antiretroviral therapy on incidence of pregnancy among HIV-infected women in Sub-Saharan Africa: a cohort study. PLoS Med.

[26] Matthews TL, Mukherjee JS (2009). Strategies for harm reduction among HIV-Affected couples who want to conceive. AIDS Behav.

[27] Jhangri GS, Heys J, Alibhai A, Rubaale T, Kipp W (2012). Unmet need for effective family planning in HIV-infected individuals: results from a survey in rural Uganda. J Fam Plann Reprod Health Care.

[28] Marlow HM, Maman S, Groves AK, Moodley D (2012). Fertility intent and contraceptive decision-making among HIV positive and negative antenatal attendees in Durban, South Africa. Health Care Women Int.

[29] Wanyenze RK, Tumwesigye NM, Kindyomunda R, Beyeza-Kashesya J, Atuyambe L, Kansiime A (2011). Uptake of family planning methods and unplanned pregnancies among HIV infected individuals: a cross-sectional survey among clients at HIV clinics in Uganda. J Int AIDS Soc.

[30] Uganda Bureau of Statistics Kampala, Uganda and MEASURE DHS ICF International Calverton, Maryland, USA (2012). Uganda Demographic Health Survey 2011. www.ubos.org/onlinefiles/uploads/ubos/UDHS/UDHS2011.pdf.

[31] Yeatman SE, Trinitapoli J (2008). Beyond denomination: the relationship between religion and family planning in rural Malawi. Demogr Res.

[32] Harrington EK, Newmann SJ, Onono M, Schwartz KD, Bukusi EA, Cohen CR (2012). Fertility intentions and interest in integrated family planning services among women living with HIV in Nyanza Province, Kenya: a qualitative study. Infect Dis Obstet Gynecol.

